# Using qualitative research to understand what outcomes matter to patients: direct and indirect approaches to outcome elicitation

**DOI:** 10.1186/1745-6215-16-S2-O39

**Published:** 2015-11-16

**Authors:** Jonathan Mathers, Thomas Keeley, Laura Jones, Melanie Calvert, Paula Williamson, Janet Jones, Christel McMullan, Susan Wright, Bridget Young

**Affiliations:** 1 University of Birmingham, Birmingham, West Midlands, UK; 2 University of Liverpool, Liverpool, Merseyside, UK

## Background

There is a need for patient involvement when selecting trial outcomes, since their priorities may differ from healthcare professionals. Qualitative research can be used to identify outcomes that matter to patients in the development of core outcome sets (COS) and in trial feasibility studies that aim to feed patient perspectives into outcome domain selection for the definitive trial. For example, the COMET database currently includes 24 published (2-3/year) and 33 ongoing COS studies utilising interviews or focus groups with patients, carers and their representatives. However, it is unclear whether direct approaches to eliciting outcomes or indirect approaches focusing on the disease and treatment are most useful in informing trial design and outcome selection.

## Findings and discussion

This presentation will consider the relative value of qualitative data collection approaches that (a) explore patients' experiential accounts of illness and treatment as a means to infer priorities for outcome domains and (b) involve explicit discussion with patients of outcome domains and preferences. Drawing on lessons from ongoing qualitative work within COS and trial feasibility studies, the presentation will consider how outcome focused discussions resonate with patients; what can be inferred about patients' outcome preferences from more expansive experiential accounts of illness and treatment; the role of researchers in interpreting these accounts and translating them to outcome domains; and the results of studies that collect both ‘types’ of data. Potential implications for COS development will be outlined.

